# Expression of an Engineered Heterologous Antimicrobial Peptide in Potato Alters Plant Development and Mitigates Normal Abiotic and Biotic Responses

**DOI:** 10.1371/journal.pone.0077505

**Published:** 2013-10-16

**Authors:** Ravinder K. Goyal, Robert E. W. Hancock, Autar K. Mattoo, Santosh Misra

**Affiliations:** 1 Department of Biochemistry and Microbiology, University of Victoria, Victoria, British Columbia, Canada; 2 Centre for Microbial Diseases and Immunity Research, University of British Columbia, Vancouver, Canada; 3 The Henry A. Wallace Beltsville Agricultural Research Center, United States Department of Agriculture, Agricultural Research Service, Sustainable Agricultural Systems Laboratory, Beltsville, Maryland, United States of America; Midwestern University, United States of America

## Abstract

Antimicrobial cationic peptides (AMPs) are ubiquitous small proteins used by living cells to defend against a wide spectrum of pathogens. Their amphipathic property helps their interaction with negatively charged cellular membrane of the pathogen causing cell lysis and death. AMPs also modulate signaling pathway(s) and cellular processes in animal models; however, little is known of cellular processes other than the pathogen-lysis phenomenon modulated by AMPs in plants. An engineered heterologous AMP, *msrA3*, expressed in potato was previously shown to cause resistance of the transgenic plants against selected fungal and bacterial pathogens. These lines together with the wild type were studied for growth habits, and for inducible defense responses during challenge with biotic (necrotroph *Fusarium solani*) and abiotic stressors (dark-induced senescence, wounding and temperature stress). *msrA3*-expression not only conferred protection against *F. solani* but also delayed development of floral buds and prolonged vegetative phase. Analysis of select gene transcript profiles showed that the transgenic potato plants were suppressed in the hypersensitive (HR) and reactive oxygen species (ROS) responses to both biotic and abiotic stressors. Also, the transgenic leaves accumulated lesser amounts of the defense hormone jasmonic acid upon wounding with only a slight change in salicylic acid as compared to the wild type. Thus, normal host defense responses to the pathogen and abiotic stressors were mitigated by *msrA3* expression suggesting MSRA3 regulates a common step(s) of these response pathways. The stemming of the pathogen growth and mitigating stress response pathways likely contributes to resource reallocation for higher tuber yield.

## Introduction

Sustained plant losses due to microbial diseases cause crop yield reduction and are of major economical concern to farmers and agriculture industry [1,2]. Throughout the world, therefore, there is an ongoing effort to develop crops resistant to different diseases. Understanding host plant-microbe interactions and elucidating mechanisms that enable some plants to defend against one or more pathogens are currently dynamic research areas [3]. The dynamics of plant response to a disease(s) change with environmental interactions [4], thus requiring an in-depth understanding of the molecular mechanisms involved. Plants that are able to resist a pathogen are more capable than the susceptible ones in creating physical barriers like thickening and lignification of the cell wall [5,6], deposit callose [7], release phenolics or toxic substances (phytoalexins, proteinases, proteinase inhibitors) that inhibit the pathogen growth or detoxify pathogen-derived toxins [8], and release chemicals that inactivate the hydrolytic enzymes secreted by the pathogen [6]. 

 Plants are known to harbor a unique systemic immunological response, which is activated upon recognition of a pathogen. One of the extensively studied inducible plant defense responses is a hypersensitive response (HR). Cells displaying HR undergo localized programmed cell death (PCD) to limit the damage, and the host plant may get immunized against subsequent pathogen attack, a phenomenon named systemic acquired resistance (SAR) [3,9]. HR is accompanied by an oxidative burst due to reactive oxygen species (ROS) [10], and changes in defense-related gene transcripts [11]. Metabolites such as glycerol-3-phosphate [12] and pipecolic acid [13] and hormones such as ethylene, salicylic acid (SA), jasmonates (JAs), nitric oxide (NO) and abscisic acid (ABA) have been implicated in plant immunity through regulating SAR [14]. Salient features of plant immunity to pathogens involve transmembrane protein receptor-like kinases (RLKs) or proteins (RLPs) [15,16], which respond to molecular patterns (pathogen associated molecular patterns – PAMPs) [17,18], as well as epigenetic-related hypomethylated genes [19]. Plants also respond to effector molecules secreted by pathogens by activating R proteins harboring nucleotide binding domain and leucine-rich repeats (NLR), leading to PCD at the infection site [3,18,20]. The NLR receptor family-triggered immunity seems conserved across plant lineages and it was suggested that NLR could interact with different host proteins to mediate distinct resistance responses [21,22]. Interestingly, expression of pepper Bs2 resistance (R) gene, which recognizes AvrBs2 effector released by *Xanthomonas* sp, was shown to provide field level resistance to the bacterial spot disease in transgenic tomatoes [23].

 Oxidative burst due to ROS generation is one of the early physiological events in plant-microbe interactions. The oxidative burst kinetics are biphasic, and the first wave might constitute a signaling function while the second wave triggering PCD [24]. That the ROS production is a feature not only restricted to HR defense but also to stress caused by abiotic factors [25], led to the studies that showed that biotic and abiotic defense responses overlap [26,27]. Notably, one of the players in the crosstalk between these two defense responses was shown to be the *abscisic acid-induced myb1* gene encoding an R2R3MYB transcription factor, which is induced by both pathogens and abiotic stresses [28]. Indirect support for crosstalks between different plant-specific defense responses was predicted from the observation of extensive overlaps in transcriptional profiles between pathogen response and wounding in *Arabidopsis* [29].

 Plants also employ another type of defense against pathogens (bacteria, fungi and viruses) through the production of antimicrobial peptides (AMPs) [30,31] that have a wide distribution from microorganisms to complex eukaryotes [32,33]. AMPs represent small proteins that vary in molecular size from 0.88 to 8.86 kDa [34] with diverse functions in innate immunity [35]. This form of defense is conserved during evolution [36]. Plant AMPs are classified into several families based on the overall charge, disulphide bonds and structural stability [34,35,37]. Their amphipathic nature provides AMPs an advantage in interacting with negatively charged microbial membrane components, and thereby altering membrane permeability of the pathogen leading to cell death [38,39]. It could place AMPs role in defense in a category different from the above mentioned HR and PCD strategy, which requires mobilization of resources and shift in metabolism to ensure plant survival [40]. However, although AMPs are believed to be anti-infective molecules bearing direct toxicity to the pathogens, it has become apparent from experiments using animal models that they modulate signaling pathway(s) and associated innate immune responses [41,42]. Thus, LL37 cationic peptide (CAP) specifically suppressed the inflammatory response to bacterial lipopolysaccharide, an important part of host defense. CAP was found to bind LPS and reduce the production of ROS by inhibiting nitric oxide (NO) synthase [43,44]. It is therefore intriguing that AMPs may play a role in cellular processes in addition to those in host defense against pathogens. In plants, information on whether HR-mediated and AMP-dependent defense responses interact with each other is scarce. Also, it is relatively unknown how plants choose one type of defense over the other. Differences notwithstanding, the plant immune response shows many parallels with animal innate immunity in terms of surveillance mechanism and HR-induced cell death [45]. The plant cell death bears resemblance with pyroptosis, a phenomenon of cell death in animal cells catalyzed by casapase-1 or with necroptosis, a mechanism involving a burst of ROS but independent of caspase activation [46].

 Potato is the fourth largest crop after rice, wheat, maize and tomato. It is one of the most important consumed vegetables in the world [47]. Diseases and pests cause major losses in total potato production, conservative estimates putting annual losses at 22% worldwide [48-50]. Late blight due to the fungal pathogen *Phytophthora infestans* [51] and bacterial soft rot and blackleg due to the bacterial pathogen *Erwinia* sps. [50] are among the major constraints to potato yield. Interestingly, potato constitutively produces AMPs, called *Snakin 1* and *Snakin 2*, whose gene transcripts are upregulated by pathogen infection and wounding [52,53]. Transgenic research has demonstrated that when heterologous antimicrobial peptide variant, synthetic AMP, or other plant AMPs are introduced into plants including potato bring about a broad-spectrum resistance to diverse types of phytopathogens [54-58]. The overexpression of potato *Snakin-1* in potato plants also enhanced resistance to *Rhizoctonia solani* and *Erwinia carotovora* [59]; however, when this gene was silenced in potato, it was found to affect growth and development processes such as cell division, primary metabolism and cell wall chemistry [60].

 Molecular engineering of the N terminus of temporin A gene, which belongs to a family of smallest antimicrobial peptides in nature, led to a new gene called *msrA3* [57]. Expression of this gene in potato led to broad spectrum resistance of the transgenic plants including the harvested tubers to two fungal and one gram negative bacterial pathogens [57]. These studies and such transgenic plants have provided a new resource for studying the effects of AMPs not only in plant pathogen response but also their impact on abiotic stress responses. In this study, we tested these transgenic potato lines for their response to abiotic stresses (induced senescence, oxidative stress and wounding) as well as to a potato pathogen (*Fusarium solani*). We show here that *msrA3*-expression modulates physiology and gene transcript profiles of the transgenic potato plants impacting HR, ROS, dark-induced senescence and wounding processes. The *msrA3*-mediated mitigation of these defense responses of potato plants was associated with a positive increase in the yield of transgenic potatoes. 

## Materials and Methods

### Plant material

Potato (*Solanum tuberosum* L.) cultivar Desiree (WT) and two transgenic lines (T3 and T26) expressing antimicrobial peptide *msrA3* gene [57] were grown in the greenhouse facility, University of Victoria, Victoria, B.C., Canada. The T3 and T26 transgenic lines represent two independent insertion events and contain a single copy of *msrA3* [57]. For brevity, leaflets of a compound leaf are referred to as leaves.

### Growth conditions and tuber yield determination

WT and the T3 and T26 transgenic plants were grown from seed tubers. Tubers with a mean weight of 56 g (50-63 g) were planted in 11-L and 15-L pots for growth chamber and greenhouse experiments, respectively. Number of plants per pot was three for growth chamber and four for greenhouse, if not specified otherwise. After 2 weeks of germination, one dose (75 g) of 6-8-6 (nitrogen/phosphorus/potash) fertilizer (Evergo Canada Inc., Delta, B.C., Canada) was applied. In growth chamber, the plants were raised under 16/8h photoperiod at 21°/18°C day/night temperature unless otherwise stated. The plants were watered as needed. At each time of planting, the pots were triplicated in a randomized clear block design in a chamber. In the greenhouse, plants were grown in 6 replications under 16/8h daylight and irrigated through automated drip irrigation. Following 16 weeks of growth, the fresh tuber yield was recorded. 

### RNA extraction and northern-blot analysis

Total RNA was isolated from frozen leaf tissue with Trizol as per the manufacturer’s protocol. RNA was fractionated on 1% agarose-formaldehyde gels and blotted onto nylon membrane (Schleicher & Schull, Germany). Gene probes were labeled with [α-^32^P] dCTP using High Prime random priming kit (Roche) and purified on ProbeQuant G-50 Micro Columns (GE Healthcare). The membranes after hybridization with the respective probes for 16h at 65°C were washed twice with 2xSSC, 0.1% SDS at 65°C for 20 min each, once in 1xSSC, 0.1% SDS and twice in 0.2xSSC, 0.1% SDS for 20 min each at 60°C. The hybridized blots were exposed to X-ray films with intensifying screens at -75°C. Following genes were analyzed: Pathogenesis-related protein (*pr-1*) (AJ250136), osmotin (osm) (AY256439), ascorbate peroxidese (*apx*) (AB041343), catalase (cat) (Z37106), γ-vacuolar processing enzyme (vpe) (D61395), senescence associated gene 12 (*sag12*) (AI776170), 13-lipoxygenase (*13-lox*) (X96406), potato peroxidase2 (*Stprx2*) (AJ401150), Cu/Zn superoxide dismutase (sod) (AF355460), longevity assurance gene1 (*lag1*) (AF198177), *rbcL* (AI486088) and glutamine synthetase-1 (*gs-1*) (AW626325). (Table S1 in File S1) lists primer sequences used for amplifying the gene probes. The genes, *pr-1*, *vpe*, *lag1*and *cat*, were PCR amplified from potato genomic DNA. For *apx* and *Cu/Zn sod*, the cDNA was prepared to RNA isolated from untreated leaves. For *Stprx2* and*13-lox*, the cDNA was prepared to RNA isolated from wounded leaves. Amplification and primer sequences for *osm*, *sag12*, *rbcL* and *gs-1* were the same as previously described [61]. cDNA was synthesized using SuperScript^Tm^II RNaseH reverse transcriptase (Invitrogen) following manufacturer’s protocol. The Qiagen MasterMix kit was used for 25 µl PCR reactions as follows: 94°C for 10 min, and 35 cycles of 94°C for 30 sec, temp (1°C below Tm of the primer sequence) for 30 sec, and 72°C for 1 min followed by 15 min extension at the end. 

### *In situ* detection and determination of H_2_O_2_

H_2_O_2_ was visualized in leaves using 3, 3’-diaminobenzidine (DAB) staining [62]. The cut end of each detached leaf was incubated with 1mg mL^-1^ DAB, pH 4.5 for 3h. After leaf de-colorization in hot ethanol (95%), the intensity of brown color stain was monitored.

For quantifying H_2_O_2_, leaf tissue (400 mg) was powdered in liquid nitrogen and then homogenized in 1 mL 10% trichloroacetic acid (TCA). The homogenate was centrifuged at 16,000 g for 15 min and supernatant collected. The content of H_2_O_2_ in the supernatant was determined by slight modification of a previously described method [63]. Briefly, the supernatant (40μL) was mixed with10 μL of 1N NaOH, and then 50μl of xylene-orange reagent (500 μM ferrous ammonium sulfate, 50 mM H_2_SO_4_, 200 μM xylene orange and 200 mM sorbitol) was added. The color was allowed to develop for 2 h and the absorbance determined at 560 nm. To ascertain that TCA addition had disabled the H_2_O_2_-metabolizing enzymes and that it was compatible with the xylene-orange assay, a known quantity of H_2_O_2_ was added during tissue homogenization for determining % recovery. The recovery was 99.2% and no activity of peroxidase or catalase was detected in the TCA-extract. The assay generated linear curves using different concentrations of H_2_O_2_ in 10% TCA. Only the relative abundance of H_2_O_2_ rather than absolute values are reported because any unknown component in the plant extract with a potential to affect the A_560_ values was not tested.

### Enzyme assays

Methods used for preparing cell-free extracts and assaying guaiacol- or pyrogallol-peroxidases activities were the same as previously described [64].

### Chlorophyll analysis

Total chlorophyll was extracted from leaves by grinding 0.5 g of tissue with 5 mL of pure acetone in a mortar with pestle followed by several extractions with 80% acetone to a final volume of 15 mL. The clarified extract was diluted, and absorbance at λ 646 nm and λ 663 nm was determined. The contents of total chlorophyll, chlorophyll *a*, and chlorophyll b were calculated as described [65].

### Determination of lipid peroxidation

Peroxidated lipids were measured as thiobarbituric acid reactive species (TBARS) [66]. Frozen leaf tissue (50 mg) was homogenized in 125 μl of 50 mM 2-morpholinoethanesulphonic acid (MES), pH 7.1, containing 2% SDS and 2 μl of 1% 2,6-di-tert-butyl-4-methylphenol (butylated hydroxytoluene). To the homogenate, 700 μl of 0.8% (w/v) thiobarbituric acid in 10% TCA was added and the contents vortexed for 1 min. The samples were heated at 95°C for 15 min, vortexed for 1 min and re-heated for 15 min. After cooling on ice, TBARS were extracted in 500 μl of *n*-butanol by vigorous mixing. The contents were centrifuged at 5,000 g for 10 min and absorbance of the supernatant was measured at 532 nm and 600 nm. TBARS content was determined after subtracting the nonspecific background absorbance at 600 nm.

### Jasmonic acid and salicylic acid analyses

Jasmonic acid (JA) and salicylic acid (SA) were analyzed at the Plant Biotechnology Institute, National Research Council, Saskatoon, Canada. The details of JA/SA extraction and analysis by High Performance Liquid Chromatography Electrospray tandem Mass Spectrometry (HPLC/ES-MS/MS) were the same as previously described [67].

## Statistical analysis

Data were statistically analyzed using analysis of variance (ANOVA), and mean separation (Tukey’s) test was performed using the SPSS statistical program.

## Results

### Delayed emergence of floral buds in transgenic potato plants expressing Msra3 gene

**Figure 1:**
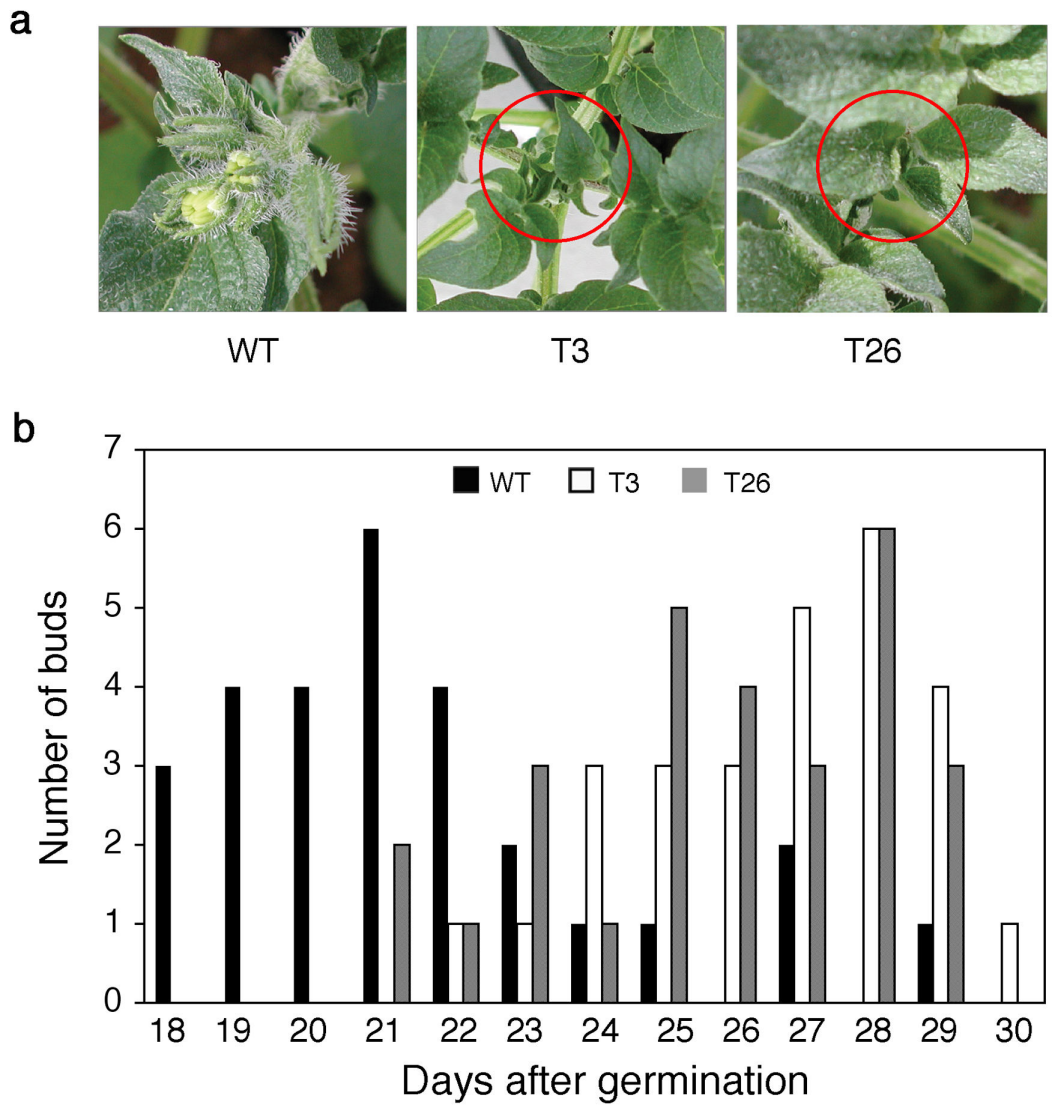
The development of floral buds is delayed in the *msr*A3-transgenics (T3 and T26). (a) Wild-type (WT) plant with the earliest emergence of a floral bud at 20 days after germination. The circles mark the terminal shoot of transgenics showing no signs of flower bud initiation. The buds were observed only on the main shoots from three replicated pots each having 3 plants grown in a growth chamber at day/night temperatures of 28^0^/22^0^C and 16/8 h light/dark cycle with light intensity of 300 µM quanta.m^-2^.s^-1^; (b) Number of newly appeared buds after indicated days of germination. The buds were counted from six replicated pots each having 4-5 plants totaling 28. The plants were grown under natural light in green house with 16 h day length, and night and day temperature varied between12-33°C.

**Figure 2:**
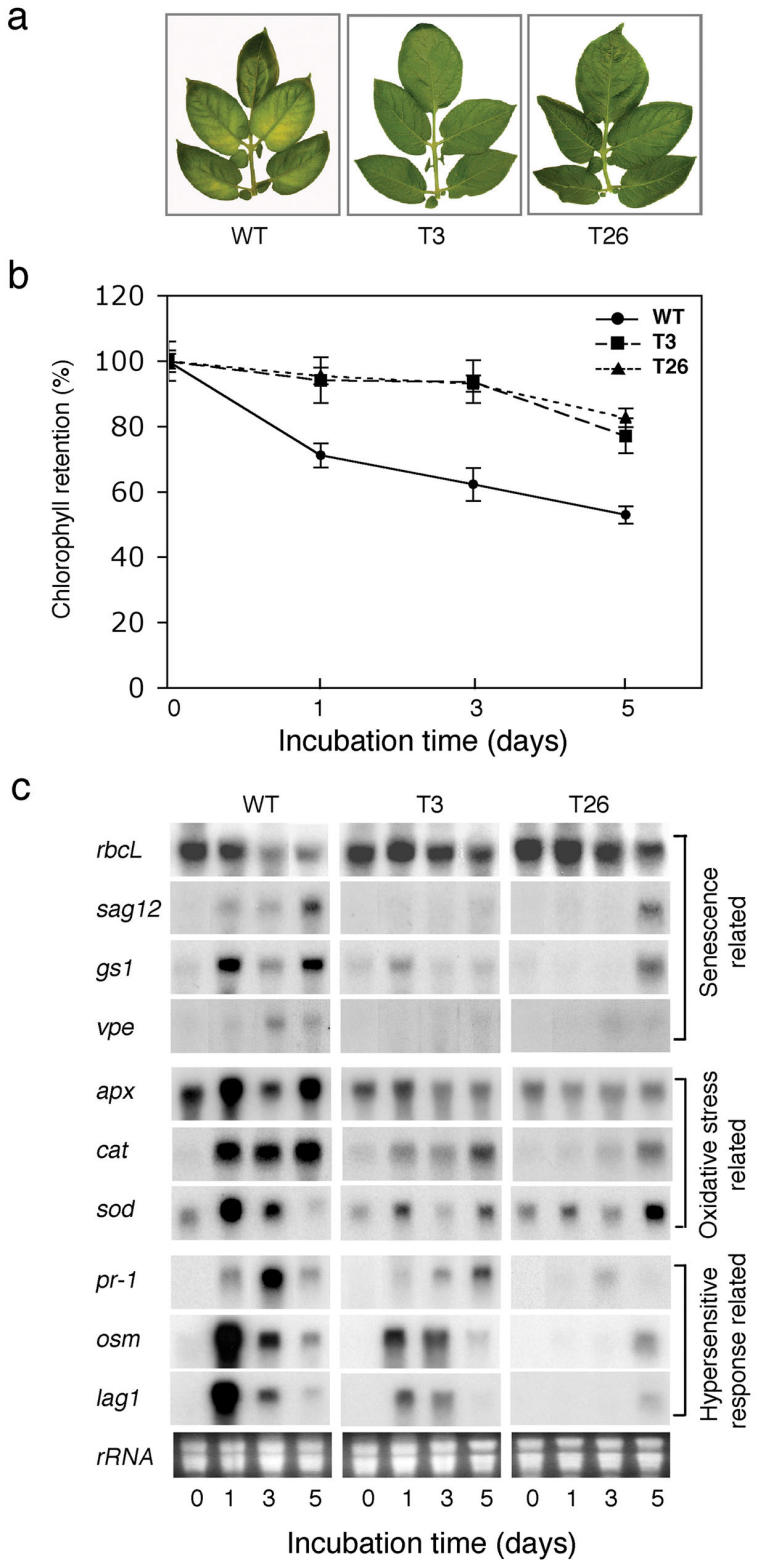
*msrA3*-transgenic plants display delay in dark-induced senescence. (a) Detached compound leaves (5^th^ to 6^th^ from top) of five week-old plants were incubated in the dark on moist filter paper in Petri dishes at room temperature and were photographed after 5 days incubation in dark. The Petri dishes were wrapped in aluminum foil to create dark conditions. The experiment was repeated at least three times and the representative results are shown here. (b) Percent retention of chlorophyll in wild-type and transgenic plants. The leaves were sampled for analysis at indicated times after incubation in darkness. Bars represent means + SE (n=3). (c) Expression profile of genes involved in HR and associated senescence in wild-type and *msrA3*-transgenic plant leaves kept in the dark (as stated in section [a]) at room temperature for the indicated times. Each lane was loaded with 20 μg of total RNA. Ethidium bromide stained rRNA served as loading control.

One of the earliest phenotypic differences observed between the wild-type (WT) and transgenic plants (T3 and T26) expressing *msrA3* (*msrA3*-transgenics) was in the development of floral buds (Figure 1a). In the growth chamber with a day/night temperature regime of 28°/22°C, floral buds in WT plants emerged at 20 days after germination while bud initiation in the two transgenic lines was not apparent by this time (Figure 1a). Fifty percent or more of the WT plants had visible buds by day 26 after germination, at least 3 days earlier than seen in transgenic plants. More validation of the delayed emergence of floral buds in the transgenic plants compared to the WT was obtained from experiments carried out in the greenhouse under natural lighting conditions with a day/night temperature variation of 33°/12°C. Under these conditions of greater fluctuations in day and night temperatures than in the controlled growth chamber, emergence of buds in the WT was accelerated (Figure 2b). In the transgenic plants, buds initiated on day 21 (T3) and day 22 (T26) of germination, by which time 60-75% of the WT plants had already developed the buds. Thus, both the transgenic lines tended to flower later than the WT plants. 

### *msrA3*-transgenics have delayed dark-induced senescence

The delay in the emergence of floral buds (flowering) in the transgenic lines indicated that *msrA3* expression may impact leaf senescence since a slower initiation of reproductive development seems associated with longer vegetative growth in some plants [68]. Therefore, we tested this possibility using the model of dark-induced senescence [69]. Detached fully developed leaves from the WT and the T3 and T26 transgenic lines were incubated at room temperature in the dark and leaf chlorophyll content was analyzed on day 1, 3 and 5. By day 5 in the dark, WT plants showed visible symptoms of senescence but the two transgenic lines were still robust and greener (Figure 2a). This difference in the physical condition of WT versus transgenics correlated with the steady loss of chlorophyll in WT leaves starting day 1 in the dark and decreasing thereafter to 50% of the original content by day 5 while in the transgenic leaves the chlorophyll content remained more or less similar until day 3 and registered a slight decline by day 5 (Figure 2b). 

### Differential expression of a select class of genes between WT and transgenic leaves during induced senescence

Delayed floral development associated with delayed dark-induced senescence in the *msrA3*-transgenics suggested that *msrA3* expression influences plant development. We therefore quantified changes in the expression of a medley of gene transcripts in the leaves of the WT and the two *msrA3-*transgenic lines including anti-senescence gene marker (large subunit of Rubisco which promotes growth) [70-72] versus pro-senescence gene markers [*senescence associated gene 12* (*sag12*) and *glutamine synthetase-1* (*gs-1*)] [73]. To that end, expression of genes not only associated with senescence [(*sag12*)*,* γ*-*vacuolar processing enzyme (vpe), (*gs-1*)] and carbon fixation [*rubisco large subunit* (*rbcL*)] but also those associated with oxidative stress [*ascorbate peroxidese* (*apx*)*,* catalase (cat)*, Cu/Zn* superoxide dismutase (sod)] and HR [*pathogenesis-related protein* (*pr-1*)*,* osmotin (osm)*, longevity assurance gene1* (*lag1*)] were analyzed by northern analysis of RNA from leaves exposed to dark induced senescence (Figure 2c). Dark-induced senescence in WT was associated with increases in the levels of *pr-1, osm, apx, cat, sod, sag12, vpe, lag1* and *gs1* concomitant with a substantial decrease in the large subunit of rubisco (*rbcL*) (panel WT). In contrast to these patterns found in WT leaves, response to dark incubation of the leaves from the two transgenics (panels T3 and T26) was different. In fact, the expression of *pr-1, osm, apx, cat, lag1* and *gs1* transcripts was mitigated in the two transgenic lines while *sag12* and *vpe* transcripts were barely observed except *sag12* in T26 after 5 days of dark incubation (Figure 2c). Notably, in the two transgenic lines, expression of *rbcL* remained at a steady level and that of *sod* fluctuated, remaining at a lower level than the WT. Thus, distinctly different patterns in the steady state levels of transcripts for genes associated with HR and senescence between WT and *msrA3*-transgenics were apparent and indicated that *msrA3* expression dampens the response seen in the WT leaves, particularly during the early phase of induced senescence (Figure 2c). 

### Higher tuber yield in *msrA3*-transgenics

In order to determine the long-term effects of *msrA3* expression in terms of the tuber yield, WT and the two *msrA3*-expressing transgenic lines were grown in three different seasons to full maturity in the greenhouse as well as in a controlled growth chamber and their tuber yield was quantified. Tuber yield was consistently and significantly (between 52-57%) higher in all the three potato lines grown in the greenhouse as compared to those grown in the growth chamber (Table 1). However, the greenhouse-grown transgenic plants yielded 15-16% more tubers than the WT, and this difference increased further to 20-27% under growth chamber conditions. The *msrA3* expression, therefore, resulted in positive phenotypic attributes that translated into higher potato productivity.

**Table 1:** Tuber yield (g/pot) of wild-type (WT) and MsrA3 transgenic plants (T3, T26).

**Season**	**Greenhouse**			**Growth Chamber**		
	**WT**	**T3**	**T26**	**WT**	**T3**	**T26**
I	810 ± 77	1030 ± 79	1020 ± 56	439 ± 52	527 ± 61	564 ± 94
II	903 ± 53	943 ± 68	960 ± 102	475 ± 48	550 ± 33	574 ± 23
III	809 ± 60	934 ± 18	954 ± 44	400 ± 29	505 ± 25	549 ± 98
**Average**	**841**	969*	978*	**442**	529**	562*

Equal number of WT and transgenic plants were grown in pots for each experiment. Tuber yield was quantified from plants with no visible symptoms of disease. Asterisks indicate significant differences comparing the transgenic lines to WT control. *p < 0.05; **p < 0.1 (Tukey’s test). Data shown are average ± s.e.m. (n = 6, greenhouse; n > 3, growth chamber.

### Basal oxidative stress in WT is mitigated in *msrA3*-transgenics during growth

**Figure 3:**
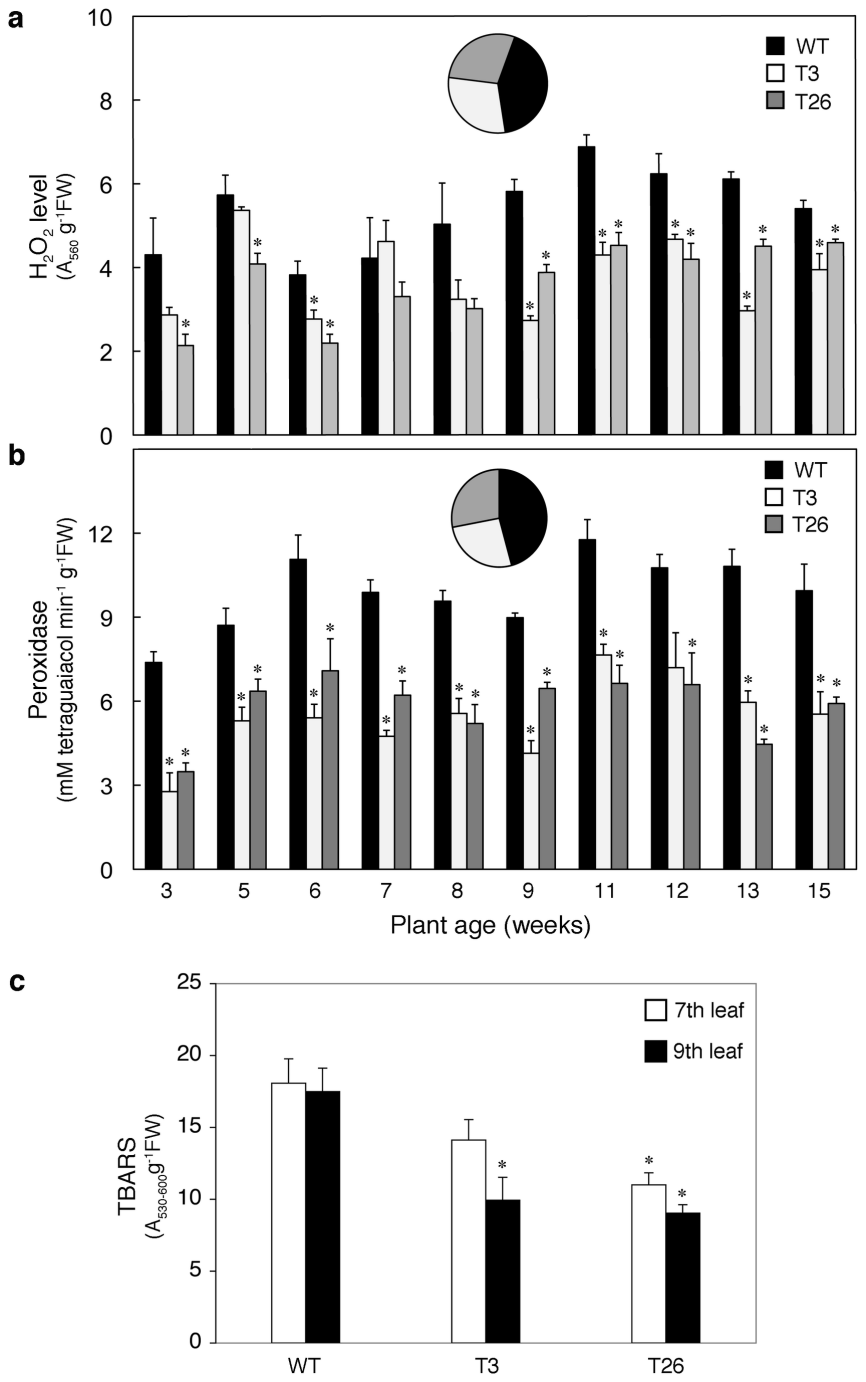
*msrA3*-transgenic plants have lower basal oxidative stress level. (a) Relative level of H_2_O_2_ and (b) Peroxidase activity at different times during the growth of wild-type and transgenic plants. For (a) and (b), the bars represent means + SE (n=3). The Pie insets represent the average distribution of data in WT, T3 and T26. (c) Lipid peroxidation measured as TBARS in wild-type and transgenic leaves. The leaves were counted from the top and sampled from 11-week old plants. Bars represent means + SE (n=4). * p < 0.05 and **p < 0.1 refer to comparison between WT and transgenic (T3 or T26) leaves.

Since certain senescence and HR responsive gene transcripts were not upregulated upon induced senescence in the transgenic lines expressing *msrA3*, as in the WT leaves, we premised that the ROS-related oxidative processes, normally connected with HR responses, may also be affected in the two transgenic lines. We, therefore, monitored the levels of ROS marker H_2_O_2_, peroxidase activity that can either generate ROS or catalyze transfer of electrons from H_2_O_2_ to the donors [74], and peroxidated lipids generated by lipid oxidation [75] in WT and *msrA3*-transgenic leaves over a period of 15 weeks. The H_2_O_2_ level in WT leaves was consistently higher than the leaves from both the transgenic lines except for the leaves of 7-week old plants (Figure 3a). On average, the H_2_O_2_ level in WT was 43-47% higher than the T3 and T26 transgenic plants. These results paralleled the peroxidase activity that was remarkably lower in the *msrA3*-transgenics than the WT; generally, peroxidase activity was 60% higher in the WT plants as compared to either of the two transgenic lines (Figure 3b). Along with reduced H_2_O_2_ levels and peroxidase activity in the *msrA3*-transgenics, thiobarbituric acid reactive species (TBARS), an index of total oxidized lipids [66], quantified in 7^th^ and 9^th^ leaf of each line was also lower in the *msrA3*-transgenics as compared to the WT (Figure 3c).

### *msrA3* expression mitigates plant response to some abiotic stresses

**Figure 4:**
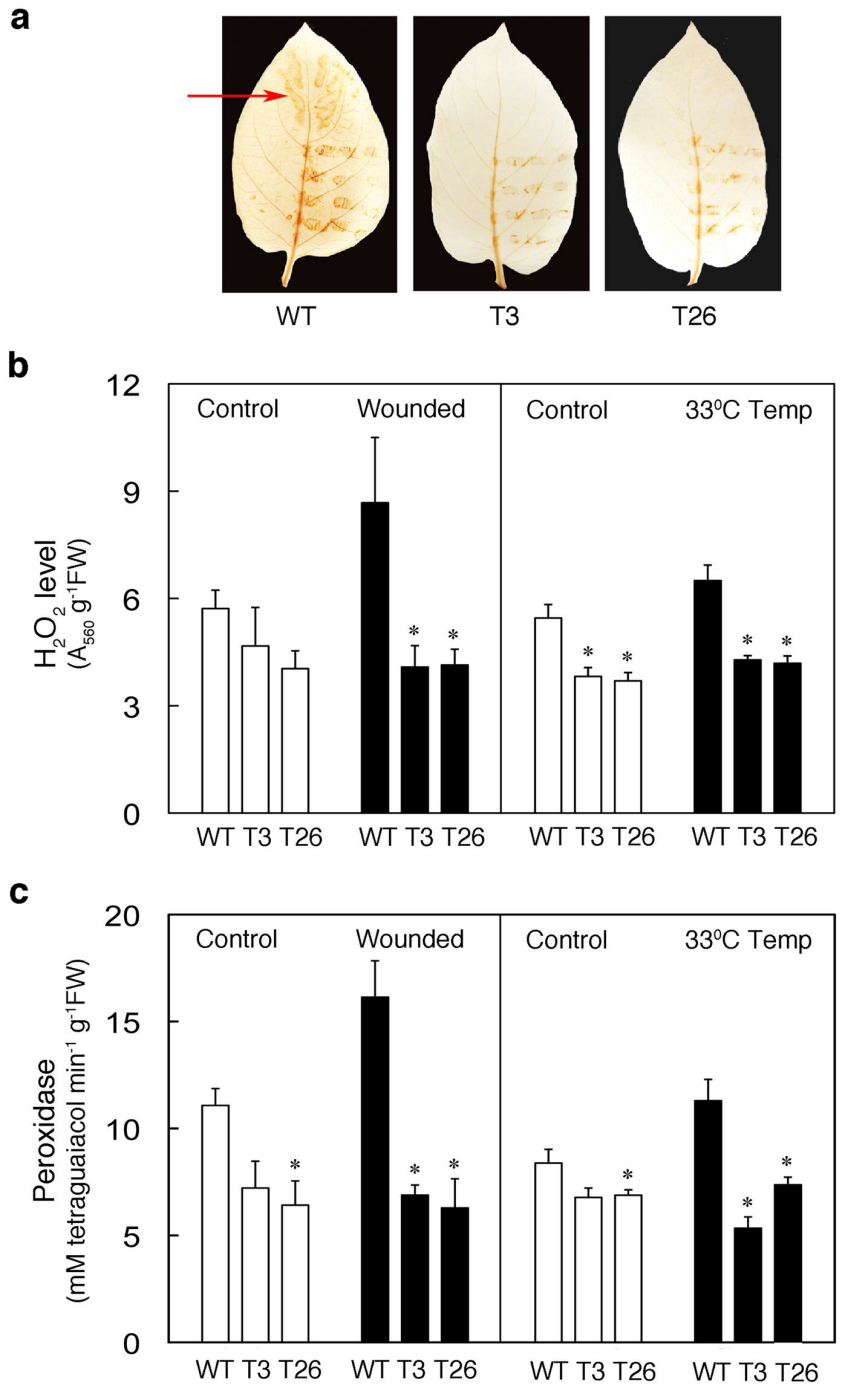
Wild-type and *msrA3*-transgenic plants display differential response to abiotic stresses. (a) Levels of H_2_O_2_ in leaves from WT and transgenic (T3 and T26) as determined by staining with DAB. Detached leaves of similar age from 5-week old plants were mechanically wounded with forceps. Lower one-half of the leaf was punctured to afflict 11-12 wounds each of approximately 2x4 mm in size. The middle vein was punctured at five places starting from the bottom. The red arrow indicates systemic wounding response. The experiment was repeated three times and the results were comparable. (b) Relative levels of H_2_O_2_ in WT and T3 and T26 leaves 1 h after wounding (Wounded) or when given temperature stress (33°C Temp) measured as described in Materials and Methods section. Bars represent means + SE (n = 4). (c) Comparison of peroxidase activity in WT and T3 and T26 transgenic leaves 1 h after wounding (Wounded) or incubation at 33°C (33°C Temp). Bars represent means + SE (n = 4)For (b) and (c), the leaves were wounded as described above in (a) and incubated on moist filter paper in Petri dishes at room temperature alongside unwounded (controls). For (b) and (c) ‘33°C Temp’, 4-week-old plants in a growth chamber at day/night temperatures of 28°/22°C and 16/8 h light/dark cycle with light intensity of 300 µM quanta.m^-2^ S^-1^ were exposed to 33°C for 6 h. The treatment was given at the beginning of the day light cycle. The control plants were kept at 28°C for the treatment period. The 6^th^ leaf from the top was excised and analyzed. * p < 0.05 and **p < 0.1 refer to comparison between WT and transgenic (T3 or T26) leaves.

Abiotic stresses [25] including wounding [76] and higher temperatures [77] are known to ameliorate basic oxidative stress in plants. We, therefore, determined wound-induced *in situ* accumulation of H_2_O_2_ by staining the leaves with 3, 3’-diaminobenzidine (DAB) after wounding [62]. As a result, the proportion of brown coloration formed around wounded areas was greater in WT leaves than those in the *msrA3*-transgenic leaves (Figure 4a). Moreover, the WT leaf developed brown coloration also in distant, unwounded parts (Figure 4a, region indicated by an arrow). Quantification of H_2_O_2_ levels in control and wounded leaves from each line verified the *in situ* visualized data. After 1h wounding, over 50% increase in the steady state level of H_2_O_2_ was observed in WT leaves, but not in the transgenic ones (Figure 4b, wounded). These data paralleled the changes in peroxidase activity upon wounding of leaves in WT plants with abrogation of the increase in *msrA3-*transgenic leaves (Figure 4c, wounded). 

 Similar response in H_2_O_2_ levels (Figure 4b, 33°C Temp) and peroxidase activity (Figure 4c, 33°C Temp) of WT and *msrA3*-transgenic leaves was observed when, instead of wounding, the leaves were given a stress of elevated temperature (33°C). 

### Patterns of *Stprx2* and *13-lox* transcripts, and content of salicylic acid and jasmonate in WT and *msrA3* **-**transgenics

**Figure 5:**
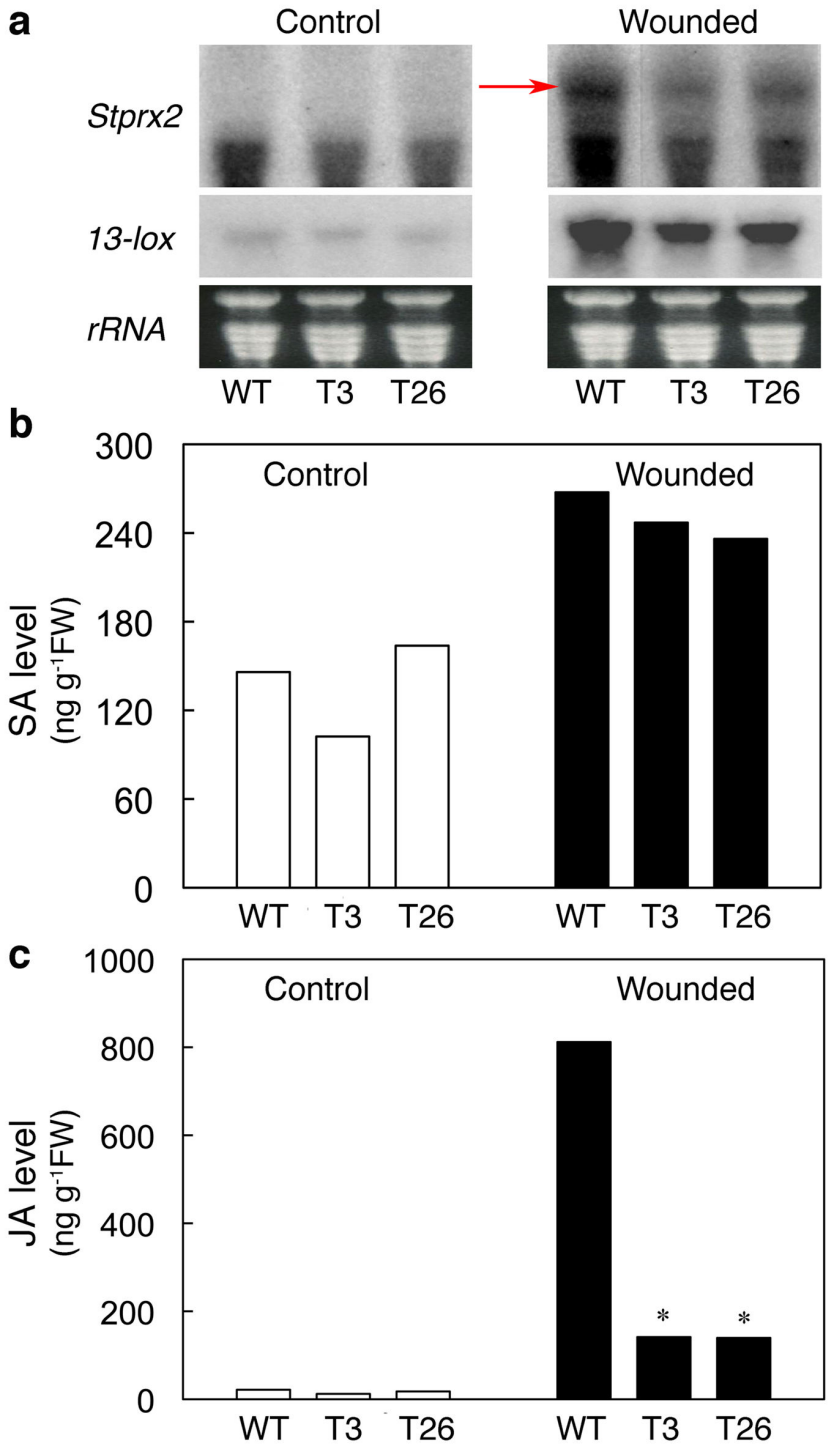
*Stprx2* expression and levels of salicylic acid (SA) and jasmonic acid (JA) in control and wounded leaves from *msrA3*-transgenic (T3 and T26) and wild-type (WT) plants. (a) Expression of *Stprx2* (indicated by arrow) and *13-lox* transcripts in WT and transgenic leaves 1 h after wounding. RNA loading was the same as described in legend to Figure 2c. (b) Levels of SA and (c) JA in WT and transgenic leaves 1 h after wounding. The values are average of two independent experiments. Wounding was carried out the same as described in the legends to Figure 4. * p < 0.05 refers to comparison between WT and transgenic (T3 or T26) leaves.

In plants, expression of peroxidase *Stprx2* [78] and *13-lox* (13-lipoxygenase) gene [79] transcripts together with increases in the levels of hormones such as salicylic acid (SA) and jasmonates (JA) are recognized as part of a wound response. To further attest the confounding effect of AMP expression in mitigating potato response to induced senescence, wounding and high temperature stress, we analyzed effects of wounding on the abundance of *Stprx2* and *13-lox* transcripts as well as the content of SA and JA in WT and *msrA3*-transgenic leaves. Wounding caused a profound increase in *Stprx2* transcripts within 1h, the increase being markedly higher in WT than the transgenic leaves (Figure 5a, *Stprx2*), which is consistent with the rise in peroxidase activity seen above (Figure 4c, wounded). Similarly, wounding led to an increased expression of *13-lox* transcripts in the WT and this increase was relatively of a lesser magnitude in the *msrA3*-transgenic plants (Figure 5a, *13-lox*).

 Wounding caused 1.5 to 2.0-fold increase in SA content across all the three lines, showing no substantial difference between WT and transgenic leaves (Figure 5b). However, the JA content increased upon wounding of both WT and transgenic leaves but the magnitude of increase was distinctly higher in the WT (about 6-fold) than the transgenics (Figure 5c). The differences in the JA content in response to wounding mimic the pattern of induction of *13-lox* transcripts (Figure 5a, *13-lox*).

### Mitigation of normal host hypersensitive response is associated with resistance to *Fusarium solani* in transgenic potato expressing *msrA3*

**Figure 6:**
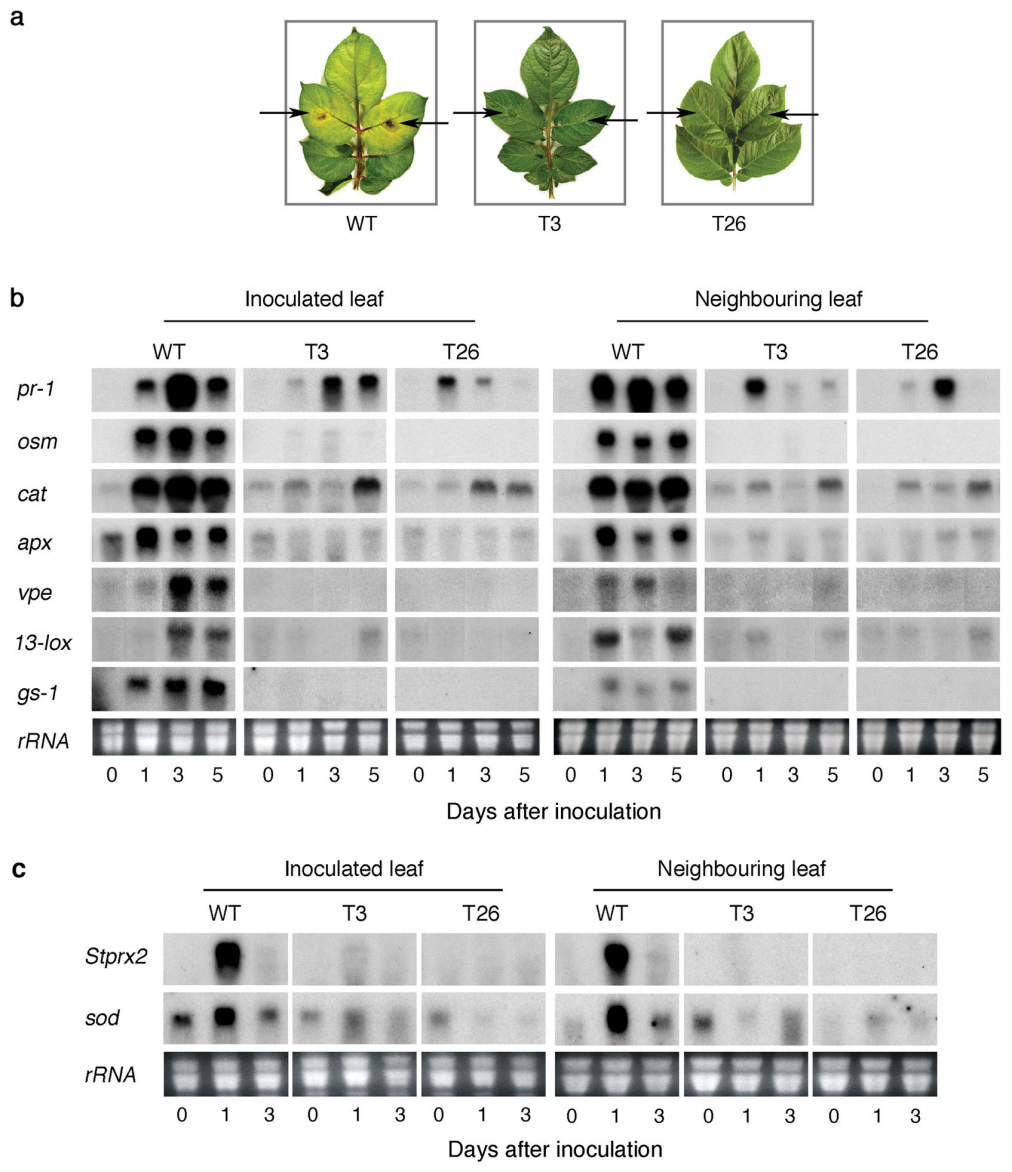
Hypersensitive response is altered in *Fusarium solani* challenged *msrA3*-transgenic plants. (a) Comparison of disease symptoms in wild-type (WT) and transgenic (T3 and T26) plants challenged with *F. solani*. Two middle leaflets of a detached compound leaf of similar age sampled from 6-7 week old plants were inoculated with 2000 conidia/leaflet (indicated by arrows), kept on moist filter paper in Petri dishes and incubated at room temperature. The conidia were collected as previously described [55]. The pictures were taken at 5 days after inoculation. (b) Expression profile of genes involved in HR at indicated times after inoculation with *F. solani* as above. (c) Expression of *Stprx2* and *sod* in WT and *msrA3*-transgenic (T3 and T26) plants at indicated days after inoculation with *F. solani*. Neighboring leaf refers to leaflets adjacent to inoculated leaflets. RNA loading was the same as described in the legend to Figure 2c.

Many of the responses of the WT to abiotic stress indicated above are also normally seen in plant response to a disease. Since, in principle, the transgenic lines developed with engineered AMPs were previously shown to be resistant to fungal and bacterial pathogens of potato [57,58], we hypothesized that pathogen response of such *msrA3-*transgenic lines may be associated with downregulated HR. Therefore, WT and AMP-transgenic leaves were challenged with *F. solani*, observed for symptom development, and then analyzed for the expression of a few marker genes associated with HR, ROS, senescence, and programmed cell death (PCD). Significant protection against symptom development to *F. solani* challenge was evident in both the transgenic (T3 and T26) leaves as reflected by the absence of necrotic lesions and the retention of greenness even on day 5 days after inoculation (dai) (Figure 6a). In contrast, the WT leaves developed necrosis on 3 dai, which became more prominent on 5 dai. The necrotic spots became chlorotic by day 5 and the progression of chlorosis was noticed also in the neighboring leaves (Figure 6a). These data confirmed previous conclusion that *msrA3* expression leads to resistance against pathogens [57].

We excised the tissue samples from a parallel set of the *F. solani*-inoculated and neighboring leaves of the WT and transgenic plants, isolated RNA and determined the patterns of gene transcripts associated with HR, ROS, senescence and PCD. Already by 1 dai, the expression of HR-related genes (*pr-1, osm*), ROS-related genes (*cat, apx*), and senescence-PCD markers (*vpe*, *gs-1*) were greatly upregulated in the inoculated WT but not in the transgenic leaves except for *pr-1* and *cat* transcripts whose induction, though small, was apparent also in the transgenic leaves (Figure 6b, compare left panel WT with T3 and T26). This early induction of the candidate genes was intensified by 3 dai and slightly declined by 5 dai for some transcripts in the inoculated WT leaves (Figure 6b, left panel WT), which corresponded to intense necrosis in such leaves. The expression of *13-lox* transcripts, responsible for the induction of defense hormone jasmonate, was evident on 3 dai in the inoculated WT leaves. In the inoculated transgenic leaves, *pr-1* and *cat* gene transcripts were present on 3 and 5 dai but their intensity was much lower than that seen in WT leaves (Figure 6b, compare WT with T3 and T26). Notably, *osm, apx, vpe, 13-lox*, and *gs-1* expression in the inoculated *msrA3* transgenic leaves (T3 and T26) was nearly absent (Figure 6b, compare lane 0 dai with lanes 1, 3 and 5 dai), excepting for a sudden appearance in *13-lox* transcript in T3 line on 5 dai but this was not reproduced with T26 line. 

A systemic response of *F. solani* challenge apparent by chlorosis of the neighboring leaves of inoculated WT plants was not seen in the *msrA3*-transgenics (Figure 6a, WT). At the level of gene transcripts, there was hardly any signal apparent for the examined genes in the neighboring leaves on 0 dai but by 1 dai all of them were induced, albeit to different extents (Figure 6b, Neighboring leaf, WT). In fact, the systemic increase in *lox-13* and *vpe* gene transcripts in WT plants occurred on 1 dai while in the inoculated WT leaves their robust expression was delayed until 3 dai. Overall, when transcript profiles of the two *msrA3*-transgenic plants (T3 and T26) were compared with WT, there was clearly a distinct absence of induction except for *pr-1* and *cat* genes (Figure 6b). 

Suppression of *cat* and *apx* gene transcripts in the two transgenics suggested a low and un-sustained HR response. This was further confirmed by analyzing the expression patterns of ROS/oxidative burst associated genes, *Stprx2* and *sod* in pathogen-inoculated and their neighboring leaves. An intense signal for their transcripts was apparent in inoculated WT and the neighboring leaves on 1 dai (Figure 6c). In the *msrA3*-transgenic lines, *Stprx2* expression was undetected while that of *sod* was shadowy. 

## Discussion

We demonstrate here that expression of an antimicrobial peptide, MsrA3, in potato provides resistance against the pathogen *F. solani*, mitigates plant defense responses including HR, ROS, leaf senescence and wounding, and alters timing of bud development, which finally culminates in increased yield of the two transgenic potato lines. Thus, while AMPs are known to be directly toxic to plant pathogens [54,57-59], as was evident here for MsrA3 potato - *F. solani* interaction, we show that *msrA3* expression also causes delayed floral development and suppresses the normal defense pathways of plants in response to a few abiotic-type stressors. 

During normal growth conditions, ROS reflected by the levels of endogenous H_2_O_2_ were generally higher in the WT than the *msrA3* transgenics and these data paralleled the total leaf lipid peroxidation status (TBARS) in WT versus transgenics. ROS levels in the WT leaves were further stimulated upon wounding as well as when the leaves were subjected to a temperature stress at 33°C. Temperature-induced stress is known to elevate H_2_O_2_ content [77]. Under both wounding and temperature stress, *msrA3* transgenics did not respond by elevating ROS levels compared to untreated samples. Elevation of ROS (measured as H_2_O_2_ content and DAB staining) in WT plants and its mitigation in the *msrA3* transgenics was associated with a parallel trend in peroxidase activity during aging, wounding and temperature stress. Thus, *msrA3* expression mitigates the WT plant ROS response to aging, wounding, and high temperature stress. 

Dark-induced senescence led to chlorophyll loss in the WT line starting at day 1 of darkness but the transgenic plants were able to retain the chlorophyll content for up to day 3 to the levels that in day 0 control. Associated with these changes was a differential accumulation of transcripts of gene markers for HR (*pr-1, osm*), ROS (*cat, apx, sod*), and senescence-PCD (*sag12*, *vpe*, *lag1*, *rbcL*, *gs-1*) in WT versus *msrA3* transgenics. A substantial up-regulation of *apx, cat*, and *sod* on day 1 of darkness in WT leaves is indicative of the onset of oxidative burst, which was associated with induction of *pr-1* and *osm* genes suggesting that HR was triggered. Relative to this WT response, the transgenics expressing *msrA3* had a subdued HR and ROS response, more subdued in T26 line than T3 line, indicating a lower oxidative stress in them. Further, *sag12*, *vpe, lag1* and *gs-1* transcripts were less abundant in the transgenics as compared to the WT, but opposite trends of accumulation were apparent for *rbcL* transcripts. These results together with differential loss of chlorophyll content and visual observations suggest that *msrA3* expression antagonizes or delays apoptosis (PCD, senescence) in transgenics compared to the WT. 

 The dampening effect of *msrA3* expression on gene markers for HR, ROS and PCD-senescence in the transgenic lines was also evident during challenge with the necrotrophic pathogen *F. solani*. MsrA3 as an antimicrobial agent effectively prevented necrosis in the leaves of transgenic potato plants in response to the pathogen challenge compared to the WT leaves. Consistent with the phenotypic observations, the transgenic leaves had subdued induction of *pr-1* and *osm* gene transcripts compared to their robust induction in the WT leaves within day 1 of pathogen inoculation. Since these genes in potato tubers form a part of hypersensitive defense response against this fungus [80], it is evident that *msrA3* expression interferes with the pathogen-mediated HR. The suppressive effect of the *msrA3* expression on HR induction was further supported by the pattern of induction or lack thereof of *cat* and *apx* gene transcripts in the transgenics. In this regard, selective activation of *vpe* and gs*-1* only in the leaves of WT plants highlights *F. solani*-mediated cell death pathway, which is clearly mitigated in *msrA3*-expressing transgenic plants. In addition to its role in senescence, *vpe* is considered as one of the architects of virus-induced HR and cell death [81]. It is worth noting that *vpe* expression was more enhanced in *F. solani* challenged WT leaves than during their dark-induced senescence. Cell death is a culmination of defense response, which is relatively more rapid and intense in response to a pathogen than during senescence. Similar trend in the activation of gene transcripts was evident in the neighboring non-inoculated leaves, which is reminiscent of the systemic response. Again, except for a subdued induction of *pr-1* and *cat* gene transcripts, expression of the remainder of the tested genes was nearly absent in the inoculated and neighboring leaves of the *msrA3* transgenic plants.

The synthetic activity of peroxidases produces O_2_^●-^, dismutated by SOD to H_2_O_2_ [82]. Induction of the potato peroxidase, *Stprx2*, which is more of an anabolic peroxidase rather than H_2_O_2_ catabolizing enzyme due to its similarity with peroxidases involved in oxidative burst [83,84] (see Figure S1 in File S1), in conjunction with *sod* transcripts during wounding and pathogen challenge in leaves of WT plants is suggestive of its involvement in oxidative burst in potato. These results also favor the possibility that *msrA3* expression intercepts normal plant defense response including ROS, HR and senescence, which in turn may contribute to the lower threshold of ROS homeostasis in the growing plants.

 Independent or co-induction of salicylic acid (SA), JA and/or ethylene is considered a common defense response of plants against pathogen attack or abiotic stressors [85-88], and likely culminates in cell death processes involving ROS. SA increases in response to biotrophs and JA in response to necrotrophs and insects [89]. Wounding induces synthesis of JA [90,91], ethylene [90,92] and SA [93]. Also, a selective involvement of JA and SA has been indicated based on the wounding agent employed [94,95]. The content of SA and JA in the unwounded and wounded leaves of WT and *msrA3* transgenic plants showed a differential pattern. SA levels were induced upon wounding to the same extent in the WT and transgenics while the JA content was considerably increased upon wounding and the wounded WT leaves contained several-fold higher JA in contrast to the wounded *msrA3* transgenic leaves. These data parallel the extent of corresponding induction of the *13-lox* gene transcripts, which are known to be involved in JA biosynthesis pathway [96]. The observed differences in the intensity of DAB-H_2_O_2_ staining in distal leaf tissue of WT and transgenics are consistent with the role of JA in systemic accumulation of H_2_O_2_ in potato, and its mitigation in plants expressing *msrA3*. 

The findings that *msrA3* expression suppresses *13-lox* transcripts during pathogen-induced HR and antagonizes wounding response of the transgenic potato plants, except may be for the induction of SA, indicate that MsrA3 interferes with JA/H_2_O_2_ signaling. Involvement of JA and SA in defense response and resistance against pathogens depend on the life style of a pathogen [97,98]. Interestingly, an increase in SA and suppression of JA, as seen here in *msrA3*-expressing potato plants, is a phenomenon known to discourage hemibiotrophic pathogens [99-101]. However, assuming that wounding during the challenge with *F. solani* would activate JA synthesis in the WT leaves as was found here upon normal wounding, we would have expected more resistance of the WT to this necrotroph, which was not found to be the case. Instead, the *msrA3* expression in the transgenics was sufficient to trigger resistance to *F. solani* even though the JA content was 1/8^th^ the level of the WT. JA and ROS are the part of a signaling network responsible for the induction of HR and, subsequently, when the cell undergoes PCD it benefits the fungus because it can feed on the dead cells and proliferate. These results demonstrate that the *msrA3* expression introduces facets of pathogen defense based on its mechanism of pathogen cell-membrane lysis while using still to be determined mechanism(s) to mitigate a number of normal host plant defense responses including wounding, high temperature and senescence. This, in turn, likely modifies bud development, prolongs vegetative phase, and tuber yield. 

The mechanism by which an antimicrobial peptide mitigates a plant’s normal response to different stresses or development is unknown. Previously, cationic antimicrobial peptides with direct microbicidal property were found to also have the ability to modify host innate immune response [41]. Nitric oxide, which mediates S-nitrosation of cellular proteins, was found to mitigate sensitivity of melanoma cells to cisplatin [102]. In another instance, negative effects of excessive N on tomato growth were mitigated by a chemical cocktail provided by a legume cover residue [103].

A stress environment induces a higher threshold of ROS, which in plants modulates development, signaling the stressed plant to grow rapidly, flower early and even shorten the grain filling period in field crops to complete the life cycle [104-107]. Such a redirection of nutrient flow from vegetative organs to reproductive growth seems to be the norm during a plant’s transition from vegetative to reproductive growth [68]. It is also known that generation of ROS-mediated HR (as a response to a stress or a pathogen attack) causes a shift in cellular metabolism for resource re-allocation [40,108], involving global changes in gene expression [109,110]. Thus, a heightened defense response of a plant contributes to the fitness cost, as seen during JA-dependent defense against herbivores [111] and pathogenesis [112,113]. In our study, the expression of *msrA3* in potato suppressed ROS (and HR) and prevented the induction of a number of gene transcripts analyzed, characteristics that were associated with an extended vegetative growth, delayed floral development, and higher tuber yield. By extrapolation to studies in the literature, we suggest that the delayed allocation of resources for reproductive growth translated into an increased tuber yield in the transgenics. Therefore, a dual action of MsrA3 involving stemming of the pathogen growth and maintaining a lower basal oxidative stress may contribute to enhanced productivity in plants. Since resource reallocation involves a global shift in the levels of hormones IAA and GA and/or nutrient balance [68], we suggest that MsrA3 function may influence these processes. 

**Figure 7:**
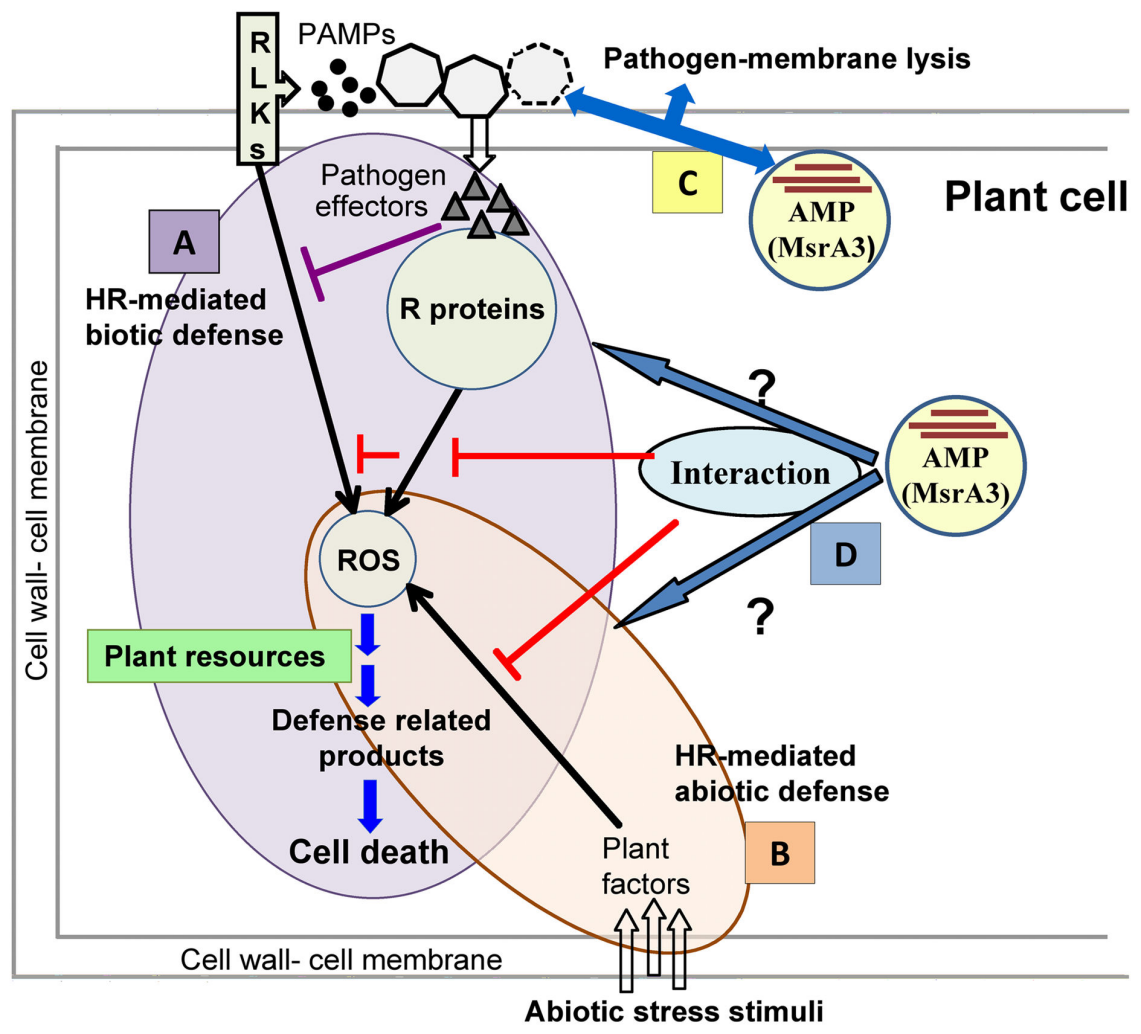
Illustration of pathways and processes in transgenic potato impacted by the expression of the antimicrobial peptide MsrA3. (A) Represents the hypersensitive response (HR) against biotic stress, outlined within a purple oval shape. Plants respond to a pathogen (heptagonals) by triggering pathogen-associated molecular patterns (PAMPs) and receptor-like-kinases (RLKs), which cause HR involving reactive oxygen species (ROS) signaling. Some pathogens secrete ‘effectors’ (shaded triangles) to suppress the PAMP-RLK mediated defense response [18]. Plants also synthesize resistance proteins (R-proteins) that recognize the effectors and induce immunity leading to ROS production [18]. The HR, often culminating in cell death, accompanies the activation of defense pathways utilizing energy and other plant resources. (B) Represents defense response against abiotic stresses, outlined within a brown oval shape bordered, where it partly overlaps with HR-mediated biotic defense. Abiotic stress stimulus is perceived by plant factors that in turn trigger the synthesis of ROS likely through the same pathway as the biotic defense response [27]. In this scenario too, stress resistance/tolerance involves activation of defense pathways, utilizing energy and other resources of the host plant. (C) and (D) Highlight MsrA3-protein defense against the pathogen causing its membrane lysis and death [38]. MsrA3 does not activate the HR and suppresses the oxidative burst (red T’s) (this paper). MsrA3 may impact other biotic and abiotic stresses (blue arrows with black boarder). By mitigating plant’s normal defense pathways, MsrA3 presence helps channel the cellular resources including energy for more growth and higher yield.

Based on the literature on plant defense responses and the findings here on the suppression of these responses by an ectopically expressed AMP, MsrA3, a working model is proposed (Figure 7). Plants respond to biotic and abiotic challenges by causing a burst of ROS that marks the induction of HR [24]. These species through a network of signaling involving NO, ethylene, JA and SA lead to comprehensive changes in gene expression responsible for the synthesis of a multitude of defense-related compounds utilizing plant resources [109]. The lack of oxidative burst, lower levels of H_2_O_2_, and early suppression of gene transcription, shown here for *msrA3* transgenics, in response to different stressors indicate that MsrA3 functions upstream of these processes. This is consistent with the suggestion that downstream the onset of stress recognition patterns the two types of stress response pathways converge [27]. Future research in this arena should throw light on the mechanisms and factors involved. Finally, the data presented here show that antimicrobial peptide-based defense (immunity) is associated with longevity of potato plants via mechanisms that bypass ROS and HR signaling. 

## Supporting Information

File S1 **Supporting Figure and Table**.
Figure S1. Amino acid sequence alignment of the potato peroxidase, StPrx2 (GenBank No. AJ401150) with peroxidases from French bean peroxidase1, FBP1 (GenBank No. 149277) and pepper, CaPO_2_ (GenBank No. DQ489711). Table S1. Primer sequences for gene probes.
(PDF)
