# Supplementary material for: Expression of an Engineered Heterologous Antimicrobial Peptide in Potato Alters Plant Development and Mitigates Normal Abiotic and Biotic Responses
Source: PLoS One. 2013 Oct 16;8(10):e77505. doi: 10.1371/journal.pone.0077505 (PMC3797780; doi:10.1371/journal.pone.0077505)
Supplement: File S1 — Supporting Figure and Table. Figure S1. Amino acid sequence alignment of the potato peroxidase, StPrx2 (GenBank No. AJ401150) with peroxidases from French bean peroxidase1, FBP1 (GenBank No. 149277) and pepper, CaPO2 (GenBank No. DQ489711). Table S1. Primer sequences for gene probes. (PDF) [file pone.0077505.s001.pdf]

SUPPORTING INFORMATION

Expression of an Engineered Heterologous Antimicrobial Peptide in Potato Affects Plant Development and Mitigates Normal Abiotic and Biotic Responses

Ravinder K Goyal[1,4], Robert E W Hancock[2], Autar K Mattoo[3*], and Santosh Misra[1]

1 Department of Biochemistry and Microbiology, University of Victoria, Victoria, British Columbia, Canada, 2 Centre for Microbial Diseases and Immunity Research, University of British Columbia, Vancouver, Canada, 3 The Henry A. Wallace Beltsville Agricultural Research Center, United States Department of Agriculture, Agricultural Research Service, Sustainable Agricultural Systems Laboratory, Beltsville, Maryland, United States of America

[4]Present address: AAFC Lethbridge Research Centre, Lethbridge, Alberta, Canada

*Corresponding author. E-mail autar.mattoo@ars.usda.gov

Figure S1. Amino acid sequence alignment of the potato peroxidase, StPrx2 (GenBank No. AJ401150) with peroxidases from French bean peroxidase1, FBP1 (GenBank No. 149277) and pepper, $CaPO_2$ (GenBank No. DQ489711). Similar amino acid residues are shaded in black and the percent similarity with StPrx2 is indicated at the end of sequences while the parentheses values indicate percent identity. The putative peptide signal sequence cleavage site is marked with a reverse triangle above the sequence. Overlines indicate four conserved disulfide bridges and dashed underlines indicate the conserved domains - peroxidase active site and proximal heme-ligand.

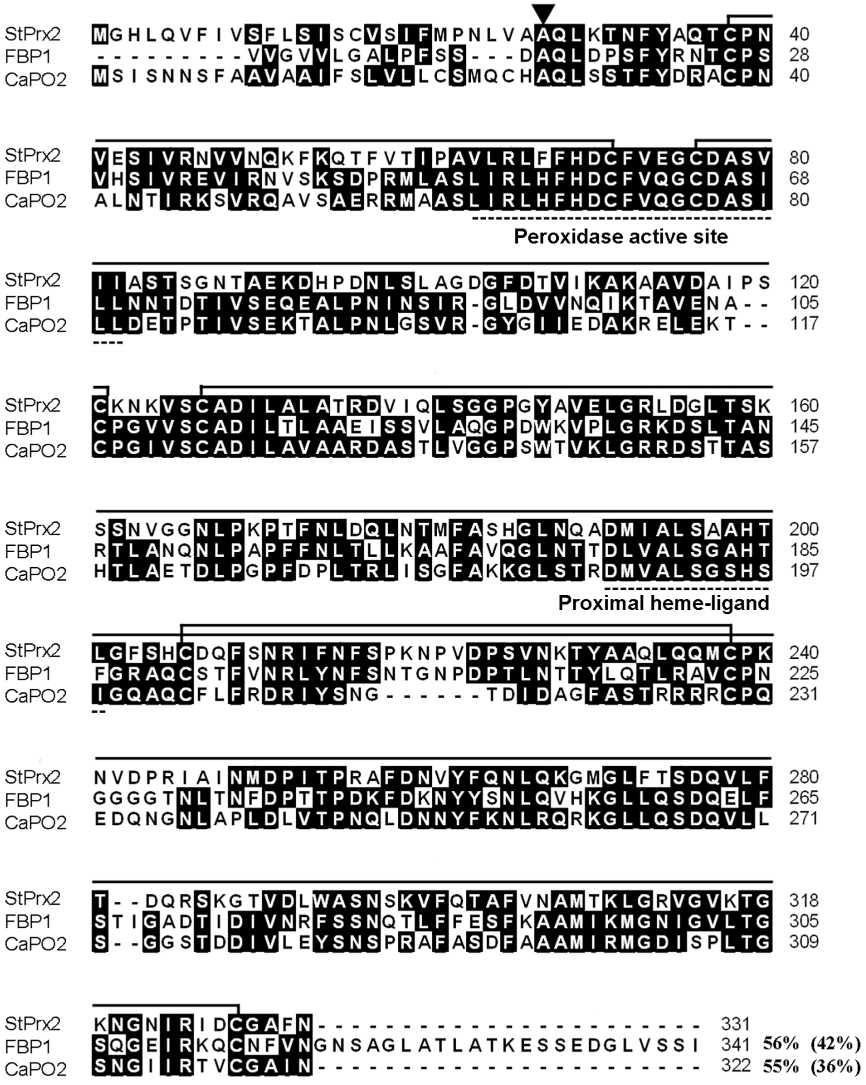

Table S1. Primer sequences for gene probes

Gene	Forward primer	Reverse primer
pr-1	5'gccatatttcactcttgtga3'	5'tcaaattaggccaaaccac3'
apx	5'tcaaggctgttgacaaatgt3'	5'agcaaatcccaattcagaga3'
cat	5'ctgttctgcttgaggattat3'	5'tctctggtgtaaaacttgac3'
vpe	5'gaaaagcaccagaaggttca3'	5'gatttttaaggcagttccag3'
13-lox	5'tatccttatgctgcaaatgg3'	5'ctagtccaccctatttatgg3'
Stprx2	5'gtcattcaactatcaggtgg3'	5'gtccatgttgatggcaattc3'
Cu/Zn sod	5'gctccaaccacagttaatg3'	5'acatagaaaacaaggctcag3'
lag1	5'cattggctaagcgtatgat3'	5'tcaaattaggccaaaccac3'
